# Supplementary material for: The commercial promotion of electronic cigarettes on social media and its influence on positive perceptions of vaping and vaping behaviours in Anglophone countries: A scoping review
Source: PLOS Glob Public Health. 2024 Jan 17;4(1):e0002736. doi: 10.1371/journal.pgph.0002736 (PMC10793929; doi:10.1371/journal.pgph.0002736)
Supplement: S2 Appendix — (DOCX) [file pgph.0002736.s002.docx]

S2 Appendix. SEARCH STRATEGIES

| **Cochrane Database of Systematic Reviews and CENTRAL** |
| --- |
| Electronic Nicotine Delivery Systems OR electronic nicotine OR Vaping OR e cig OR ecig OR electronic cigarette OR electronic nicotine OR nicotine and vape OR vaping  AND  Marketing OR advertising OR direct-to-consumer advertising OR commercial OR advert OR Promot  AND  Social media OR social medias OR web 2 OR blog OR wiki OR wikis OR weblog OR web log OR facebook OR face book OR tweet OR tweets OR tweeting OR twitter OR you tube OR youtube OR social web OR social software OR social medium OR crowd sourcing OR crowdsourcing OR instant messaging OR microblogging OR social bookmark OR patientslikeme OR patient forum or discussion forum OR online forum OR chat forum OR chatforum OR chatroom OR chat room OR online adj2 discussion OR discussion board OR online chat OR online adj2 communication OR digital media OR digital medias OR personal health message OR user comment OR patients posts OR users posts OR internet accounts OR internet sites OR message board OR web scale OR google plus OR user generated OR consumer generated OR online health content OR internet narrative OR social network OR online social network OR social networking OR online comment OR internet forum OR web forum OR internet media OR web media OR sentiment analysis OR Blogging OR Crowdsourcing |
| **Epistemonikos** |
| (title:(Electronic Nicotine Delivery Systems) OR abstract:(Electronic Nicotine Delivery Systems)) OR (title:(electronic cigarette) OR abstract:(electronic cigarette)) OR (title:(electronic cigarettes) OR abstract:(electronic cigarettes)) OR (title:(e-cigarettes) OR abstract:(e-cigarettes)) OR (title:(e-cigarette) OR abstract:(e-cigarette)) OR (title:(vaping) OR abstract:(vaping)) OR (title:(electronic nicotine) OR abstract:(electronic nicotine)) OR (title:(e cig) OR abstract:(e cig)) OR (title:(nicotine) OR abstract:(nicotine)) AND (title:(marketing) OR abstract:(marketing)) OR (title:(social marketing) OR abstract:(social marketing)) OR (title:(promotion) OR abstract:(promotion)) OR (title:(advertising) OR abstract:(advertising)) OR (title:(advertizing) OR abstract:(advertizing)) OR (title:(advertisement) OR abstract:(advertisement)) AND (title:(social media) OR abstract:(social media)) OR (title:(facebook) OR abstract:(facebook)) OR (title:(face book) OR abstract:(face book)) OR (title:(instagram) OR abstract:(instagram)) OR (title:(twitter) OR abstract:(twitter)) OR (title:(you tube) OR abstract:(you tube)) OR (title:(youtube) OR abstract:(youtube)) OR (title:(blog) OR abstract:(blog)) OR (title:(web) OR abstract:(web)) OR (title:(tik tok) OR =*abstract:(tik tok)) OR (title:(tiktok) OR abstract:(tiktok)) OR (title:(internet) OR abstract:(internet)) OR (title:(web media) OR abstract:(web media)) OR (title:(digital media) OR abstract:(digital media)) |
| **MEDLINE** |
| 1     Electronic Nicotine Delivery Systems/  2     electronic nicotine.ti,ab,ot,kf,kw.  3     Vaping/  4     e cig*.ti,ab,ot,kf,kw.  5     ecig*.ti,ab,ot,kf,kw. (164) 6     electronic cigarette*.ti,ab,ot,kf,kw.  7     electronic nicotine.ti,ab,ot,kf,kw.  8     (nicotine and vap*).ti,ab,ot,kf,kw.  9     vaping.ti,ab,ot,kf,kw.  10     or/1-9 (10347) 11     marketing/  12     advertising/  13     direct-to-consumer advertising/  14     social marketing/  15     marketing.ti,ab,ot,kf,kw.  16     commercial*.ti,ab,ot,kf,kw.  17     advert*.ti,ab,ot,kf,kw.  18     Promot*.ti,ab,ot,kf,kw.  19     or/11-18  20     10 and 19  21     social media.ti,ab,kw.  22     social medias.ti,ab,kw.  23     web 2*.ti,ab,kw.  24     blog*.ti,ab,kw.  25     wiki.ti,ab,kw.  26     wikis.ti,ab,kw.  27     weblog*.ti,ab,kw.  28     web log*.ti,ab,kw.  29     facebook.ti,ab,kw.  30     face book.ti,ab,kw.  31     tweet.ti,ab,kw.  32     tweets.ti,ab,kw.  33     tweeting.ti,ab,kw.  34     twitter*.ti,ab,kw.  35     you tube.ti,ab,kw.  36     youtube.ti,ab,kw.  37     social web.ti,ab,kw.  38     social software.ti,ab,kw.  39     social medium.ti,ab,kw.  40     crowd sourcing.ti,ab,kw.  41     crowdsourcing.ti,ab,kw.  42     instant messaging.ti,ab,kw.  43     microblogging.ti,ab,kw.  44     social bookmark*.ti,ab,kw.  45     patientslikeme.ti,ab,kw.  46     patient forum*.ti,ab,kw.  47     discussion forum*.ti,ab,kw.  48     online forum*.ti,ab,kw.  49     chat forum*.ti,ab,kw.  50     chatforum*.ti,ab,kw.  51     chatroom*.ti,ab,kw.  52     chat room*.ti,ab,kw.  53     (online adj2 discussion*).ti,ab,kw.  54     discussion board*.ti,ab,kw.  55     online chat*.ti,ab,kw.  56     (online adj2 communication*).ti,ab,kw.  57     digital media.ti,ab,kw.  58     digital medias.ti,ab,kw.  59     personal health message*.ti,ab,kw.  60     user comment*.ti,ab,kw.  61     patients posts.ti,ab,kw.  62     users posts.ti,ab,kw.  63     internet accounts.ti,ab,kw.  64     internet sites.ti,ab,kw.  65     message board*.ti,ab,kw.  66     web scale.ti,ab,kw.  67     google plus.ti,ab,kw.  68     user generated.ti,ab,kw.  69     consumer generated.ti,ab,kw.  70     online health content.ti,ab,kw.  71     internet narrative*.ti,ab,kw.  72     social network* site*.ti,ab,kw.  73     online social network*.ti,ab,kw.  74     social networking.ti,ab,kw.  75     online comment*.ti,ab,kw.  76     internet forum*.ti,ab,kw.  77     web forum*.ti,ab,kw.  78     internet media.ti,ab,kw.  79     web media.ti,ab,kw.  80     sentiment analysis.ti,ab,kw.  81     Social Media/  82     Blogging/  83     Crowdsourcing/  84     or/21-83  85     20 and 84 |
| **Embase** |
| 1     Electronic Cigarette/  2     electronic nicotine.ti,ab,ot,kf,kw.  3     Vaping/  4     e cig*.ti,ab,ot,kf,kw.  5     ecig*.ti,ab,ot,kf,kw.  6     electronic cigarette*.ti,ab,ot,kf,kw.  7     electronic nicotine.ti,ab,ot,kf,kw.  8     (nicotine and vap*).ti,ab,ot,kf,kw.  9     vaping.ti,ab,ot,kf,kw.  10     or/1-9  11     marketing/  12     advertizing/  13     direct-to-consumer advertizing/  14     social marketing/  15     marketing.ti,ab,ot,kf,kw.  16     commercial*.ti,ab,ot,kf,kw.  17     advert*.ti,ab,ot,kf,kw.  18     Promot*.ti,ab,ot,kf,kw.  19     or/11-18  20     10 and 19  21     social media.ti,ab,kw.  22     social medias.ti,ab,kw.  23     web 2*.ti,ab,kw.  24     blog*.ti,ab,kw.  25     wiki.ti,ab,kw.  26     wikis.ti,ab,kw.  27     weblog*.ti,ab,kw.  28     web log*.ti,ab,kw.  29     facebook.ti,ab,kw.  30     face book.ti,ab,kw.  31     tweet.ti,ab,kw.  32     tweets.ti,ab,kw. 33     tweeting.ti,ab,kw.  34     twitter*.ti,ab,kw.  35     you tube.ti,ab,kw.  36     youtube.ti,ab,kw.  37     social web.ti,ab,kw.  38     social software.ti,ab,kw.  39     social medium.ti,ab,kw.  40     crowd sourcing.ti,ab,kw.  41     crowdsourcing.ti,ab,kw.  42     instant messaging.ti,ab,kw.  43     microblogging.ti,ab,kw.  44     social bookmark*.ti,ab,kw.  45     patientslikeme.ti,ab,kw.  46     patient forum*.ti,ab,kw.  47     discussion forum*.ti,ab,kw.  48     online forum*.ti,ab,kw.  49     chat forum*.ti,ab,kw. 50     chatforum*.ti,ab,kw.  51     chatroom*.ti,ab,kw.  52     chat room*.ti,ab,kw.  53     (online adj2 discussion*).ti,ab,kw.  54     discussion board*.ti,ab,kw.  55     online chat*.ti,ab,kw.  56     (online adj2 communication*).ti,ab,kw.  57     digital media.ti,ab,kw.  58     digital medias.ti,ab,kw.  59     personal health message*.ti,ab,kw.  60     user comment*.ti,ab,kw.  61     patients posts.ti,ab,kw.  62     users posts.ti,ab,kw.  63     internet accounts.ti,ab,kw.  64     internet sites.ti,ab,kw.  65     message board*.ti,ab,kw.  66     web scale.ti,ab,kw.  67     google plus.ti,ab,kw.  68     user generated.ti,ab,kw.  69     consumer generated.ti,ab,kw.  70     online health content.ti,ab,kw.  71     internet narrative*.ti,ab,kw.  72     social network* site*.ti,ab,kw.  73     online social network*.ti,ab,kw.  74     social networking.ti,ab,kw.  75     online comment*.ti,ab,kw.  76     internet forum*.ti,ab,kw.  77     web forum*.ti,ab,kw.  78     internet media.ti,ab,kw.  79     web media.ti,ab,kw.  80     sentiment analysis.ti,ab,kw.  81     Social Media/  82     Blogging/  83     Crowdsourcing/  84     or/21-83  85     20 and 84 |
| **PsycInfo** |
| 1 Electronic Cigarettes/  2 electronic nicotine.mp.  3 Vaping/  4 e cig*.mp.  5 ecig*.mp.  6 electronic cigarette*.mp.  7 electronic nicotine.mp.  8 (nicotine and vap*).mp.  9 vaping.mp.  10 or/1-9  11 marketing/  12 advertising/  13 direct-to-consumer advertising/  14 social marketing/  15 marketing.mp.  16 commercial*.mp.  17 advert*.mp.  18 Promot*.mp.  19 or/11-18  20 10 and 19  21 social media.mp.  22 social medias.mp.  23 web 2*.mp.  24 blog*.mp.  25 wiki.mp.  26 wikis.mp.  27 weblog*.mp.  28 web log*.mp.  29 facebook.mp.  30 face book.mp.  31 tweet.mp.  32 tweets.mp.  33 tweeting.mp.  34 twitter*.mp.  35 you tube.mp.  36 youtube.mp.  37 social web.mp.  38 social software.mp.  39 social medium.mp.  40 crowd sourcing.mp.  41 crowdsourcing.mp.  42 instant messaging.mp.  43 microblogging.mp.  44 social bookmark*.mp.  45 patientslikeme.mp.  46 patient forum*.mp.  47 discussion forum*.mp.  48 online forum*.mp.  49 chat forum*.mp.  50 chatforum*.mp.  51 chatroom*.mp.  52 chat room*.mp.  53 (online adj2 discussion*).mp.  54 discussion board*.mp.  55 online chat*.mp.  56 (online adj2 communication*).mp.  57 digital media.mp.  58 digital medias.mp.  59 personal health message*.mp.  60 user comment*.mp.  61 patients posts.mp.  62 users posts.mp.  63 internet accounts.mp.  64 internet sites.mp.  65 message board*.mp.  66 web scale.mp.  67 google plus.mp.  68 user generated.mp.  69 consumer generated.mp.  70 online health content.mp.  71 internet narrative*.mp.  72 social network* site*.mp.  73 online social network*.mp.  74 social networking.mp.  75 online comment*.mp.  76 internet forum*.mp.  77 web forum*.mp.  78 internet media.mp.  79 web media.mp.  80 sentiment analysis.mp.  81 Social Media/  82 Blogging/  83 Crowdsourcing/  84 or/21-83  85 20 and 84 |
| **Science Citation Index** |
| TS=(Electronic Nicotine Delivery Systems or electronic nicotine or Vaping or e cig* or ecig* or electronic cigarette* or electronic nicotine or nicotine and vape or vaping)  AND  TS=(marketing or advertising or direct-to-consumer advertising or commercial or advert* or Promot*)  AND  TS=(social media  or social medias  or web 2  or blog  or wiki  or wikis  or weblog  or web log  or facebook  or face book  or tweet  or tweets  or tweeting  or twitter  or you tube  or youtube  or social web  or social software  or social medium  or crowd sourcing  or crowdsourcing  or instant messaging  or microblogging  or social bookmark  or patientslikeme  or patient forum  or discussion forum  or online forum  or chat forum  or chatforum  or chatroom  or chat room  or online adj2 discussion  or discussion board  or online chat  or online adj2 communication  or digital media  or digital medias  or personal health message  or user comment  or patients posts  or users posts  or internet accounts  or internet sites  or message board  or web scale  or google plus  or user generated |
