# Supplementary material for: The commercial promotion of electronic cigarettes on social media and its influence on positive perceptions of vaping and vaping behaviours in Anglophone countries: A scoping review
Source: PLOS Glob Public Health. 2024 Jan 17;4(1):e0002736. doi: 10.1371/journal.pgph.0002736 (PMC10793929; doi:10.1371/journal.pgph.0002736)
Supplement: Attachment — Submitted filename: Response to the Reviewers.docx [file pgph.0002736.s003.docx]

**Response to the reviewers:**

Dear reviewers,

We would like to thank you for your valuable time, expertise and feedback on our manuscript peer review process. Please find our responses below.

**Response to Reviewer #1:**

**Reviewer comment:** The authors may consider (briefly) introducing the regulatory framework of E- Cigarettes, specifically with regard to advertising on social media platforms (either in the introduction or in a table), as this provides context for both, the thematic analysis as well as policy discussion that follow.

**Authors answer:** Thank you for this suggestion. We agree that introducing a regulatory framework of Electronic Cigarettes, specially related to its advertising on social media helps to contextualize the thematic analysis and policy implications for this scoping review. Hence, we have added in the introduction section (pg no: 3) , a brief statement with regards to the most relevant Framework Convention on Tobacco Control (FCTC), developed by the World Health Organization.

**Reviewer comment**: To improve clarity, the authors may consider defining ‘social media’ at the onset even prior to the “inclusion and exclusion” criteria (pg no:7,8).

**Authors answer:** Thank you – we have added a definition to the introduction (pg no: 3)

**Reviewer comment**: It may also be interesting for readers to understand the basis for selecting ‘core’ Anglophone countries for the SR. Are the authors aware of the status of research studies in other geographies as well?

**Authors answer:** Your comment is extremely pertinent, thank you. We have included in the methods (pg no: 7), a definition of Anglophone countries, as well the basis for limiting this Scoping Review setting. Given the current state of research on Electronic Cigarettes across the globe, it is necessary to justify the geographical limitation of this Scoping Review. This criterion was based on data availability and language accessibility of publications related to e-cigarettes originated from countries with English as the primary language. Furthermore, the influence of Anglophone countries over global regulatory discussions concerning e-cigarettes, correlates to increased research funding for health studies, policy advocacy, and international cooperation. While recognizing that the promotion of e-cigarettes in social media is a global public health concern, this scoping review findings is able to provide significant insights and discourses on the topic which extend to non-Anglophones regions.

**Reviewer comment**: The thematic analysis culminates in a host of interesting issues that demand further analysis. With respect to the ‘policy and practice implications’ (pg no:26), authors may explain how their findings support stricter regulations? This section would benefit from illustrations and a bit more analysis. For example, it would be good to understand or even get a better sense of what it means to have strict regulations (mentioned in the conclusion as well).

**Authors answer:** Thank you for your helpful comment, we agree that a more refined policy and implications section is necessary due to the complexity of the thematic analysis. For clarity, we have extended the concept of strict regulations, also reinforced in the conclusion. Despite acknowledging the necessity for a more concise definition of strict regulations and how this scoping review support them, we would like to endorse that providing a comprehensive policy implication is not the primary purpose this scoping review, but to contribute to the development of further research on the topic based on the gaps of included studies.

**Reviewer comment**: Is it possible to address influencers/celebrities’ sponsorship via regulation (think about jurisdiction and other challenges associated with regulating and monitoring social media) or do we need to think of other innovative, non-regulatory approaches.

**Authors answer:** We agree that addressing the sponsorship of celebrities and social media influencers a requires more attention. Hence, we added in the policy implication section (pg no: 27) a more detailed explanation. We appreciate your comment.

**Reviewer comment**: The 11 articles reviewed have a geographical focus, does the US have ‘strict(er)’ marketing regulations, or have other countries been able to do a better job regulating this issue?

**Authors answer:** Thank you for your comment. For comparative purposes, we added both on the introduction (pg no: 3) and policy and implication section (pg no: 28), comparisons of e-cigarettes regulations.

**Response to the Reviewer #2:**

**Reviewer comment for the introduction**: It may be worthwhile to mention the conflict of interest of some actors involved in e-cigarette policy debates. Particularly, how tobacco companies have created e-cigarette products, or invested in them (for example, JUUL).

**Authors answer:** We appreciate this suggestion. We have introduced a brief statement on tobacco industry involvement in the e-cigarette market and concern regarding industry involvement in policy debates related to the e-cigarettes to the introduction (pg no 3, 4) and to the policy and implication section (pg no: 27, 28).

**Reviewer comment for the introduction**: Citation error, third paragraph, first sentence.

**Authors answer:** Thank you, the error is corrected.

**Reviewer comment for the introduction**: Context on regulatory requirements of vaping advertisers, or social media requirements of advertisers, would helpfully contextualize how these activities occur.

**Authors answer:** We agree that introducing a regulatory framework of Electronic Cigarettes, specially related to its advertising on social media helps to contextualize the thematic analysis and policy implications for this scoping review. Hence, we have added in the introduction section (pg no: 3), a brief statement with regards to the most relevant Framework Convention on Tobacco Control (FCTC), developed by the World Health Organization.

**Reviewer comment for the methodology**: I have concerns over the review methodology. Particularly, the inclusion/exclusion criteria established. I believe the strategy may miss relevant articles because of the decision to exclude user-generated content/unclear application of how content/thematic are decided for inclusion. A rationale is needed to explain why non-commercial promotion of electronic cigarettes was excluded (non-sponsored posts, user generated content). Often times advertisements are not declared on social media. Regulatory agencies have warned many companies for not declaring ads. Notably, vaping products are found to not declare their ads as ads, or be compliant with standards (for example, <https://www.ncbi.nlm.nih.gov/pmc/articles/PMC9869128/>). Other studies, such as those assessing user-generated content, find at times commercial origins or undertones (for example, <https://gh.bmj.com/content/7/6/e009112>). This search strategy seemingly would not capture such activities.

**Authors answer:** Thank you for your valuable feedback and concerns with regards to this scoping review methodology. We appreciate the opportunity to address these comments and improve the quality of this work. Contributing to a more defined and concise research objective, we decided to limit this scoping review to industry-generated promotion. User-generated content is a complex phenomenon, and by addressing it, additional sources of influence (such as peer-to-peer influence [Association of e-Cigarette Advertising, Parental Influence, and Peer Influence With US Adolescent e-Cigarette Use | Public Health | JAMA Network Open | JAMA Network](https://jamanetwork.com/journals/jamanetworkopen/fullarticle/2796834)), would need to be considered and precisely analysed. We are aware that user-generated content can carry undertones of commercial affiliations, which are not always explicitly declared. However, in the absence of disclosed paid relationships, user-generated content may be interpreted as organic posts, and consequently, exempt of advertising restrictions. Hence, it is crucial to mention that this exclusion criteria were thoughtfully based on our focus on the industry as e-cigarettes promoters. Nonetheless, further research is necessary to cover user generated content. Despite this we have acknowledged the impact of excluding user generated content in the “limitations of this scoping review” section, we recognize the need to explore those decisions early on the manuscript aiding transparency. To improve clarity, we provided a clear concept of user generated content and how it resonates on e-cigarettes regulations on social media in the methods section (pg no: 7).

**Reviewer comment for the methodology**: Similarly, why were some content or thematic analysis studies excluded? This type of research is a major contributor to understand how health-harming products are portrayed online to social media users. It is unclear the difference between the content/thematic studies excluded, versus those included. A rationale/explanation would be helpful.

**Authors answer:** We understand your concern, and we would like to clarify our rationale for this exclusion. We agree that conducting a thematic/content analysis is a much-needed contribution to understanding the promotion of electronic cigarettes on social media. Considering the research objective, merely including descriptive analysis of e-cigarettes commercials content on social media may fail to address its impact on users’ favourable perceptions and vaping behaviours. Therefore, a deliberate emphasis was placed on thematic/content analysis studies assessing e-cigarettes advertisements and its engagement metrics, represented by users’ comments, shares, reposts, retweets, or likes. Since users’ engagement could be translated as endorsements of the promoted content, these metrics are integral components of this study. This selection criteria aimed to cover how effectively e-cigarette adverts resonated with the social media audience, ultimately shaping users’ perceptions and potentially influencing vaping behaviours. To improve the clarity of the methodology, we extended this justification in the “selecting the studies sections” (pg no: 7, 8).

**Reviewer comment for the discussion**: Reference error, page 23 first paragraph, last sentence.

**Authors answer:** Thank you, the error is corrected.

**Reviewer comment for the discussion**: May be worthwhile to explore how vaping compares to other health-harming industry commercial activities on social media. Is vaping unique? Broader issues of social media or vaping-specific regulatory absences?

**Authors answer:** Thank you! We added on the policy and implications section (pg no: 27, 28), a brief statement of how the alcohol industry compares to e-cigarettes promotion on social media.

Once again, we appreciate your valuable feedback!
